# General Dementia Training for the Social Care Workforce: A Systematic Review

**DOI:** 10.1111/jan.70288

**Published:** 2025-10-21

**Authors:** Clarissa Giebel, Thais Caprioli, Marie Poole, Catherine Talbot, Jacqui Cannon, Kerry Hanna, Louise Robinson, Deborah Rozansky, Hilary Tetlow, Madeleine Walpert, Mark Gabbay

**Affiliations:** ^1^ Department of Primary Care & Mental Health University of Liverpool Liverpool UK; ^2^ NIHR Applied Research Collaboration North West Coast Liverpool UK; ^3^ Population Health Sciences, Newcastle University Newcastle upon Tyne UK; ^4^ NIHR Applied Research Collaboration North East and North Cumbria Cumbria UK; ^5^ Department of Psychology Bournemouth University Bournemouth UK; ^6^ Lewy Body Society Wigan UK; ^7^ School of Allied Health Professions and Nursing University of Liverpool Liverpool UK; ^8^ Social Care Institute for Excellence London UK; ^9^ SURF Liverpool Liverpool UK; ^10^ Dementia UK London UK

**Keywords:** dementia, social care, training

## Abstract

**Aim:**

The aim of this systematic review was to assess and synthesize the global evidence on existing general dementia training and education for the social care workforce.

**Design:**

Mixed‐methods systematic review.

**Data Sources:**

Systematic searches on five databases (PubMed, APA PsychINFO, CINAHL Plus, Scopus, Web of Science) were conducted for articles published between 2010 and July 2024.

**Methods:**

Each abstract and full text was screened by two research team members, with conflicts of inclusion dissolved by a third team member. Data were extracted and studies narratively synthesized by the group into comparisons of content, delivery mode, workforce and outcomes/impact.

**Results:**

Twenty‐seven studies from 13 mainly high‐income countries were included in this review. Most studies provided training to care home staff, with studies using remote, in‐person and blended training delivery modes. While the focus was on generic dementia education, various interventions have been evidenced in the social care workforce, to different effects. Most changes in outcomes were reported for staff knowledge and confidence, while evidence on impacts on people with dementia is limited and mixed.

**Conclusion:**

There are various types of in‐person and remote dementia training available for the social care workforce, with overall positive impacts on knowledge and change in care delivery. Evidenced interventions need to be implemented across countries and have the potential to improve dementia knowledge, particularly in lower‐ and middle‐income countries where evidence and the social care workforce are limited.

**Implications for the Profession and/or Patient Care:**

Findings provide clear recommendations on the value and benefit of diverse dementia training on the social care workforce, care delivery and limited but emerging evidence on service user outcomes. Nurses are key parts of the staff working in social care settings, including care homes and would thus benefit from the identified dementia training.

**Patient or Public Contribution:**

Two former unpaid carers and three voluntary sector staff helped interpret the findings and reviewed drafts of the manuscript. They are co‐authors.

## 1 Introduction

Receiving support and care with performing everyday activities including dressing, feeding, washing and preparing a meal will at one stage become vital for people diagnosed with dementia (Giebel et al. 2015). Whilst a substantial amount of care is provided by family members and friends in an unpaid capacity (Carers UK 2021), social care workers are crucial to support the person with dementia to live as independently as possible—either in their own home or in a residential care facility.

Qualifications for social care vary substantially across countries, with limited to no social care oftentimes available in lower‐ and middle‐income countries (LMICs) such as Colombia, Uganda or India. Research from eight European countries has shown how professionals providing diagnosis, treatment and care for people with dementia tend to be educated at Bachelor level or above, whilst those providing everyday care, such as washing and dressing, have substantially lower levels of training or no formal training (Hallberg et al. 2014). In lower‐ and middle‐income countries, dementia care is limited if non‐existent in many parts, mostly relying on health care professionals to provide a diagnosis only without social care afterwards to fill the necessary care and support gap (Breuer et al. 2021).

Many families affected by dementia, as well as social care workers themselves, express a lack of dementia knowledge in paid carers who are looking after relatives with dementia, including those providing home care, day care and residential care (Jones et al. 2013; Barbosa et al. 2014; Polacsek et al. 2019; Leverton et al. 2021). Limited targeted training can lead to poorer quality of care (Gilster et al. 2018), so that educating the social care workforce about the basics of dementia is crucial. Specifically, a lack of workforce knowledge about dementia has emerged as a key cause of inequalities in dementia care (Stephan et al. 2018), as also depicted in the Dementia Inequalities Model (Giebel 2024). By providing targeted dementia‐specific training to the workforce, one root cause of receiving unequitable outcomes in care for this disease could thus be addressed.

With a recognition of a lack of adequate and consistently implemented dementia training in social care, there is a growing evidence base surrounding dementia‐specific training for the social care workforce (Prahl et al. 2016; Torres‐Castro et al. 2022). Albeit an influx of more targeted dementia training in recent years, there has been no systematic synthesis of the existing evidence in recent years. One systematic review from 2017 included studies on any type of training and education surrounding dementia, not only basic dementia knowledge, and explored the training delivery and impacts in both health and social care workforces. Including over 150 studies, Surr et al. (2017) reported that the most effective interventions included face‐to‐face participation, were designed with an underpinning theory, and lasted at least 8 h in total. Spector et al. (2016) included 16 studies reporting on any type of training interventions (not dementia specific) for staff working in residential care or assisted living facilities, having searched only two databases in 2013. Whilst the review reported on staff outcomes in dementia care, only one study was included that reported on a quasi‐experimental evaluation of a dementia knowledge intervention for care staff (Featherstone et al. 2004). Another review from the same year looked at dementia education specifically; however, it only delivered to staff working in hospital settings and not in social care settings (Scerri et al. 2017). Thus, it appears that to date no review has focused on general dementia education interventions for social care staff only, despite the need for an up‐to‐date overview of dementia education in the workforce also to inform the new UK Social Care Commission (report expected in 2028).

Therefore, the aim of this systematic review was to evaluate the evidence on existing generic dementia education for the social care workforce, to understand what interventions exist for improving capacity and training in the social care workforce on dementia and how effective these are. Generic dementia training was defined as focusing on general aspects of the condition, such as its neurodegeneration, associated symptoms (including cognitive, behavioural, speech and motor functioning changes) and different types of dementia, instead of focusing on specific issues such as managing pain in dementia or the physical care provision of people with dementia.

## 2 Methods

The review protocol was prospectively registered on PROSPERO [ID: CRD42024564308]. The Preferred Reporting Items for Systematic Reviews and Meta‐Analyses (PRISMA) guidelines were followed when conducting the review and reporting the results (Page et al. 2021).

### 2.1 Search Strategy

A total of five databases were searched (PubMed, APA PsychINFO, CINAHL plus, Scopus, Web of Science) in July 2024. A search restriction was applied to exclude studies published prior to 2010 and those written in languages other than English and German. The principle of Setting, Population, Intervention, Control and Evaluation (SPICE) (Booth 2006) was applied while devising the search strategy. The search terms (Table 1) were searched within the titles and abstracts of studies across the five databases. Additional manual searches were conducted. The reference list of similar reviews and those of the included studies within this review were screened.

**TABLE 1:** Search terms.

Setting	Population_1	Population_2	Intervention
Social care service	Dementia	Social care provider	Dementia training
‘care home’; ‘nursing home’; respite; ‘home care’; ‘domiciliary care’; ‘residential care’; ‘day care’; ‘day centre’; ‘day center’; ‘peer support’; ‘home adaptation’; ‘social care’; ‘community‐based care’; ‘long‐term care’; ‘care and support’	Dement*; Alzheimer*; ‘ADRD’	Staff; workforce; manager*; personnel; assistant*; practitioner*; provider*; ‘work force’; ‘paid caregiver’; ‘formal caregiver’; nurse; ‘care navigator*’; ‘link worker*’	Development; train*; ‘capacity building’; program*; intervention*; education; module*; e‐learning; learning

### 2.2 Study Selection

#### 2.2.1 Inclusion and Exclusion Criteria

Studies were included if published between 2010 and 2024; published in English or German; primary studies focusing on the delivery of a dementia training for the employed social care workforce. The reason for including studies published from 2010 onwards was to consider that the role of technology in education and how learning is delivered and accessed has changed substantially in the past 15 years.

Articles were excluded if they did not report on new primary data collection (such as conference abstracts, editorials, letters, reviews, not peer‐reviewed journal articles); focused on specific care elements of dementia, such as pain management, behavioural management, Namaste care or other forms of personalised care; or included health care (not social care) workforce. Studies focusing on students were excluded.

#### 2.2.2 Screening

The titles and abstracts of retrieved records were assessed by two research team members against inclusion criteria in Stage 1. This task was shared among five researchers, ensuring that each title and abstract was assessed by two reviewers (CG, TC, CT, MW, MP). The selected records were read in full text in Stage 2 independently by two researchers (CG, TC, CT, MW, MP), and articles that met the inclusion criteria were included in the review. Any discrepancies at Stages 1 or 2 were resolved in discussion with the wider research team. We used Rayyan to conduct the review, which is an online web‐based software that facilitates systematic review screening, including duplicate removal.

### 2.3 Quality Assessment

The methodological assessment of included papers with a mixed methods or quantitative study design was assessed with the Mixed Methods Appraisal Tool (MMAT) (Hong et al. 2018), and papers with a qualitative study design were assessed with the Critical Appraisal Skills Programme (CASP) checklist for qualitative research (CASP 2024). The MMAT comprises two screening questions followed by five questions specific to the study design employed. The CASP checklist for qualitative research comprises nine questions. Questions on both tools are rated by reviewers with ‘yes’, ‘no’ or ‘can't tell’. Two reviewers independently assessed the methodological quality of each included paper. Any discrepancies were discussed and resolved within the wider research team.

### 2.4 Data Extraction and Synthesis

Data from the included studies were extracted by two research team members (CG, TC), including information on country, sample characteristics, type of study, focus of intervention, delivery mode of intervention and effectiveness of intervention. Data were synthesised by at least three research team members (CG, TC, MP) with a formal narrative synthesis approach. This involved grouping studies together based on different criteria, which were generated during full text screening and data extraction, with studies evaluated based on the findings, methods, strengths and limitations and comparing the findings across different studies with similar methods or interventions. Both quantitative and qualitative findings were summarised and synthesised.

## 3 Results

### 3.1 Overview of Included Studies

**FIGURE 1:**
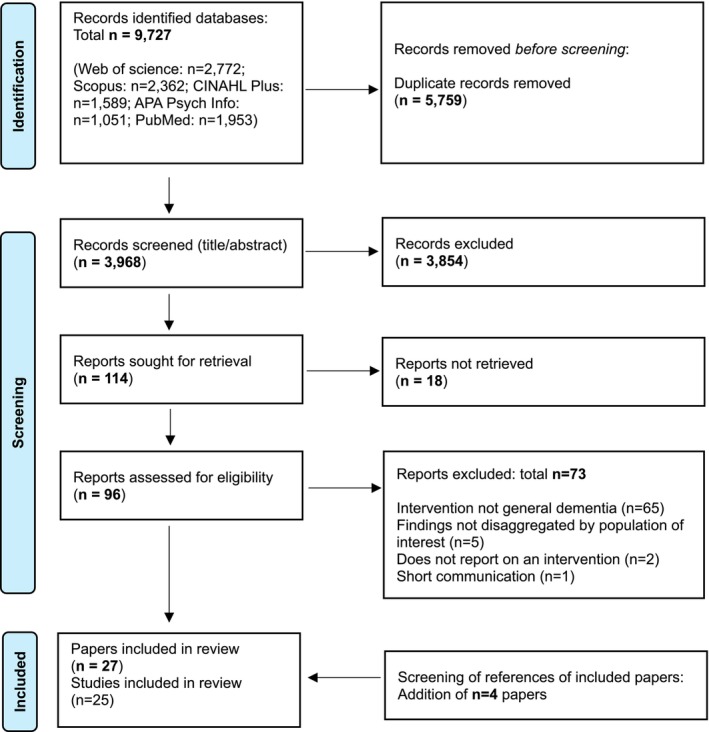
PRISMA Flowchart of included studies.

From 96 full texts screened, 27 papers (25 studies) were included in this review. Figure 1 shows the PRISMA flowchart of included and excluded articles. Evidence from 13 countries was included (Australia, Canada, China, Hong Kong, Malta, Mexico, Norway, Portugal, Sweden, Taiwan, Uganda, UK, USA). Apart from two upper‐middle‐income countries (China and Mexico) and one lower‐income (Uganda) country, evidence mostly stemmed from high‐income countries. Of the 27 included papers, the majority were conducted in the USA (*n* = 6; 22.2%) and UK (*n* = 6; 22.2%), followed by China (*n* = 3; 11.1%). Most papers employed a mixed‐method design (*n* = 13; 48.1%), followed by a quantitative (*n* = 9; 33.3%) and qualitative (*n* = 5; 18.5%) approach. Six (22.2%) papers included a control group. Most interventions were delivered by in‐person approaches and were addressed to staff working in care homes. Table 2 provides further details on the key characteristics of each included paper, and below, evidence is grouped and narratively compared based on content, delivery method, type of workforce (i.e., care home, home care or across mixed care settings) and intervention outcomes.

**TABLE 2:** Overview of included studies.

Authors (years)	Country	Care setting	Population	Study design	Intervention/training		
					Focus	Delivery mode	Frequency/duration
Aicken et al. (2021)^a^	England	*n* = 2 nursing home	*n* = 14 HCA undertook training; *n* = 2 interviewed. *n* = 1 nurse; *n* = 1 manager interviewed, did not attend training.	*Qualitative* Semi‐structured interviews	Stimulate the experience of having dementia (three scenarios: a street setting; a shop; making refreshments for visitors at home)	*Online* Smart phone App with VR. Accessed in nursing home	Encouraged to try at least two scenarios, lasing under 30 min
Beer et al. (2011)^b^	Australia	*n* = 18 residential care facilities	*n* = 1072 residential care facility staff agreed to participate; *n* = 326 attended one or more of the 94 sessions; *n* = 117 completed all sessions. Feedback collected from 93 sessions and 1067 evaluations received. *n* = 22 dementia champions identified; *n* = 16 completed entire programme. *n* = 3 FGD (*n* = 6 or 7) with dementia champions; and *n* = 2 FGD (*n* = 10 each) with residential care facility staff managers.	*Mixed method* Process evaluation. Recorded participation rates, closed and open‐ended questionnaires (feedback following each learning encounter) FGDs	Comprised 27 lessons relating to: communication; responding to pain, delirium and depressions; working effectively with residents; apply and understand how positive values underpins QoL for all.	*In person* In each care setting Identified dementia champions were offered a facilitator's role in the education programme. Introductory and final workshop for dementia champions and facility managers	Lessons divided into 30‐min blocks. Blocks could be built into sessions of varying lengths of time to suit each facility. Ranged from 1.5 to 7 h.
Chan et al. (2020)	Hong Kong	Community care centres; day care centres; residential care homes	*n* = 17 health and social care professionals were trained as trainers *n* = 1347 staff members participated; *n* = 1264 completed pre‐post assessments (*n* = 195 facilitators and *n* = 1069 learners)	*Mixed method* Questionnaire (DKAS; DAS; SNCW; SCIDS) baseline and 12‐months follow‐up Reflexive essays	Promote holistic care. Topics included: dementia and persons with dementia; person‐centred care and building meaningful relationship; communication and behaviours; support for people with dementia, family and carers; health and wellbeing; and legal aspects and issues related to dementia	*In person* In each care setting	Three‐day facilitator workshop. Each facilitator provided 12 two‐hour training sessions to a group of around six staff, over 6 months.
Cooper et al. (2024)	England	*n* = 6 home care agencies (*n* = 4 intervention; *n* = 2 control)	*n* = 63 HCW (*n* = 44 in intervention; *n* = 19 control) baseline; *n* = 46 follow‐up (*n* = 30 intervention; *n* = 16 control) *n* = 16 caring dyads (*n* = 13 intervention (*n* = 7 received intervention); *n* = 3 control) baseline; *n* = 10 caring dyads (*n* = 7 intervention; *n* = 3 control) follow‐up Proxy measures completed: intervention *n* = 19 (*n* = 7 cares; *n* = 12 HCW); control *n* = 5 (*n* = 3 carers; *n* = 2 HCW)	*Quantitative* Cluster randomised single blind feasibility trial Questionnaires (WRSI; SCIDC; DEMQOL‐proxy; DAD; NPI‐Q; adapted version of the client services receipt inventory; home care satisfaction measure) at baseline and post‐intervention 6 months Health economic analysis.	NIDUS professional: Sessions comprised importance of peer support and HCW wellbeing; building positive relationships and managing reluctance to engage; supportive people stay active/involve meaningful activities; teamworking; quality care and developing individual and agency action plans. Agency managers sessions covering topics likely to influence agency and adoption of NIDUS professional NIDUS Family: Delivered to the carer/as caring dyads. Identified personalised and measurable goals and selected modules to help achieve goals.	*Online* Manualised training delivered by non‐clinical facilitators. Facilitators received 1 h group supervision fortnightly from a clinical psychologist. 3 months implementation practice.	NIDUS professional: 6 months 6 videocall sessions (1–1.5 h) over 3 months, then monthly for 3 months Session groups of 6–8 HCWs Monthly groups to support HCW apply learning in practice. HCWs who missed sessions, invited to 1:1 catch up sessions. Intervention arm agency managers invited to 3 individual sessions NIDUS Family: 6–8 sessions over 6 months
Dobbs et al. (2018)	USA	*n* = 10 nursing homes	*n* = 48 certified nursing assistants	*Mixed method* Questionnaire (Programme‐specific: questions on confidence, knowledge, skills.) Open‐ended questions feedback training	CARES ADL programme comprises 10 modules on PCC: connecting with residents; assessing the problem, responding appropriately, evaluating staff response and sharing the results of the evaluation with other staff members.	*Online*	Designed as 10× 1‐h modules, with a 40 minimum threshold, took participants 7‐17 h (mean: 10.4 h) Able to complete training at own pace
Ehlman et al. (2018)	USA	*n* = 12 skilled health facilities	*n* = 24 skilled nursing facility health workers participated *n* = 22 completed pre‐post‐test (*n* = 8 registered nurses & licensed practical nurses; *n* = 3 social service staff; *n* = 3 activities/life enrichment staff; *n* = 2 administration; *n* = 6 others (medical director; special care unit directors and therapists))	*Quantitative* One group pre‐post test (programme specific: questions on knowledge and attitudes/perceptions)	Teepa snows' positive approach to care trainer‐coach certification Day 1: retained versus lost neurological abilities; sensory input and processing; progression of dementia to learn how individuals are unique and capable; unmet emotional and physical needs; care partnering techniques. Day 2: rehearsed trainer or coach knowledge/skills, adaptations for learning styles, personality traits	Blended – both in‐person and online	7 h online pre‐work training. A two‐day onsite workshop
Fallahpour et al. (2020)	Sweden	*n* = 1 home care agency	*n* = 34 nursing assistants working in home care; *n* = 30 follow‐up; *n* = 21 participated in pre and post follow‐up assessments	*Quantitative* Pre‐post test (SDCS; CCQ) Pre and post interventions at 12 months	Aimed to translate guidelines to evidence‐based practice, using guidelines to develop, implement and evaluate practice and disseminate improvement processes and findings among staff at home care centre Evidence‐based care and PCC	*In person* Seminars, presentations, poster presentations, workshops Group activities facilitated by faculty members from Karolinska Institute with expertise in caring for persons with dementia In‐person discussions	12 months Seminar on Swedish national guidelines care of persons with dementia (4 h); workshop discuss workplace SWOT in relation to guidelines (4 h); 10 home‐based improvement seminar, one facility‐wide day, one facility wide post session Participants worked in 5 small groups (6–7 per group) and met 10 times over a year.
Figueiredo et al. (2013)	Portugal	*n* = 1 nursing home	*n* = 6 staff participated; *n* = 5 attended FGD *n* = 6 residents with dementia	*Mixed method* Video recording of residents' behaviour during care FGD	Increase knowledge regarding dementia care, promote skills to integrate motor and multisensory stimulation in daily care and develop coping strategies to manage emotional work‐ related demands.	*In person* At the care home Handouts provided Group discussions, home‐work exercises, role‐playing and brainstorming were used during the psychoeducational session Delivered by a multidisciplinary team	8 psychoeducational sessions, lasting 90 min, were held fortnightly Participants individually assisted while providing care by a gerontologist and the physical therapist—in the 3 days following each psychoeducational session
Hobday, Savik, and Gaugler (2010)	USA	*n* = 8 nursing homes	*N* = 63 DCWs approached; *n* = 34 completed pre and post questionnaires	*Mixed method* Pre‐post‐test questionnaire (programme specific: knowledge, confidence; feedback training) Open‐ended questions (feedback training)	Develop efficient training for staff on late‐stage dementia care. 3 modules: introduction to dementia; rethinking activities and toileting	*Online* Accessible from computer	3 modules included 3 h content. Asynchronous training approach
Hobday, Savik, Smith, and Gaugler (2010) and Hobday, Savik, and Gaugler (2010)	USA	*n* = 3 nursing homes; *n* = 1 assisted living facility	*n* = 49 CNA participated; *n* = 40 completed pre‐post questionnaire of dementia care knowledge; *n* = 35 completed feedback questionnaire	*Mixed method* Pre‐post‐test questionnaire (programme specific: knowledge, confidence; feedback training) Open‐ended questions (feedback training)	CARES programme To educate CNA on issues related to dementia, including introduction to dementia (assessment and staging); behaviour management; food and fluid intake; pain management; communicating with residents.	*Online* Web‐based (narrated, text, video and interactive exercises)	Each module included approximately 45–70 min of content. CNAs took approximately 60 min to complete each module.
Inker et al. (2021)	USA	*n* = 9 nursing homes	*n* = 481 staff activated microlearning; *n* = 250 participated; *n* = 190 completed pre‐training questionnaire; *n* = 130 completed post‐training questionnaire. *n* = 26 pre‐post training questionnaires able to be linked (technical issue) *n* = 250 (*n* = 97 direct care staff with supervisory responsibility; *n* = 49 direct care staff with no supervisory responsibility; *n* = 8 director of nursing; *n* = 53 support staff; *n* = 12 activities staff; *n* = 8 social workers; *n* = 11 admin/executive director)	*Mixed method* Questionnaire Pre‐post training (DAS; NHNAJSQ; training feedback) Microlearning metric data Open‐ended questions: training feedback Post‐intervention Interviews with managers perceived success; feedback training	Five topics: meeting people with dementia where they are; living with dementia; listening and speaking; actions and reactions; being with a person with dementia: approaches; you make a difference. Following feedback, added working with families and self‐care. Topics built upon each other, but they could also stand alone. Certificates if completed all lessons	*Online* Accessed on computer, tablet or smartphone Nursing homes encouraged to identify ‘super users’ to act as champions—encouraging peers to complete training/assist with trouble shooting	52 lessons averaged 6 min content A new lesson a week. Flexible access. Nursing home received weekly flyers highlighting lessons Participants received weekly reminders to complete lessons
Irvine et al. (2012)	USA	*n* = 1 LTC	*n* = 68 NDCWs (*n* = 25 nurses/LPNs; *n* = 43 non‐licensed) participated in training *n* = 57 NDCWs completed all assessments	*Quantitative* Pre‐test post test *n* = 2 baseline questionnaire 1 month interval and a post‐test questionnaire 14 days post training. Questionnaires (programme‐specific: video‐situation testing, items addressing psychosocial constructs associated with behaviour change and measures training‐acceptance)	Topics covered: Speaking Skills, Reacting Skills, Redirection, Communication Cards, and When Bad Things Happen	*Online* Video modelling vignettes, right way and wrong way exemplars, testimonials and narration Browsable website. Could leave or return at will to any section	Designed to require about 2 h of seat time to complete all modules, but use‐time could vary depending on user interests (e.g., not viewing or repeating content) or pace of use
Karungi et al. (2022)	Uganda	Community based support	*n* = 30 LHW	*Qualitative* Pre‐post training interviews	Competency domains and skills: understanding dementia; community‐based management and care for people with dementia; community engagement; and monitoring and evaluation	*In person* Delivered by a psychiatrist	5‐day intervention; 8 weeks implementation
Kelleher et al. (2022)	England	*n* = 1 home care agency	*n* = 8HCW completed baseline; *n* = 7 commenced intervention; *n* = 5 completed all 6 training sessions (*n* = 4 HCW; *n* = 1HCW supervisor). Of which *n* = 2 attended 2 implementation sessions and *n* = 2 attended one session. *n* = 5 interviewed post training sessions; *n* = 3 interviewed post implementation session Interviewed homecare manager and facilitators	*Mixed method* Interviews and group interview post‐training (3 months) and following implementation sessions (6 months) Facilitator reflexive logs	Focuses on exploring practical changes to support client's independence and to develop self‐care strategies to manage job stresses. Implementation period to facilitate application of learning into practice, share challenges and/or success, access peer support, group problem‐solving and discuss practical application of the training.	*Online* Via video call	Six session manualised training programme Group sessions for 6‐8homecare workers lasting 60–75 min 3‐month implementation period. Facilitators met monthly with the group
Kelleher et al. (2024)	UK	*n* = 4 home care agencies	Intervention: *n* = 44 HCW; *n* = 13 client dyads recruited; *n* = 29 HCW; *n* = 4 client dyads completed. Qualitative data: *n* = 20 HCW; *n* = 3 managers *n* = 3 family carers	*Mixed method* Proportions eligible HCWs receiving any intervention; number of sessions attended; intervention fidelity (observational fidelity checklist, programme specific); adherence of clients and carers. Feedback on sessions by email, interviews and FGD with HCWs, managers and family carers	Same as Cooper et al. (2024)	*Online* Same as Cooper et al. (2024)	Same as Cooper et al. (2024)
Larocque et al. (2014)	Canada	*n* = 1 LTC facility	*n* = 11 staff (*n* = 4: personal support worker; *n* = 1 registered practice nurse; *n* = 1 registered nurse; *n* = 1 administrator; *n* = 1 life enrichment; *n* = 1 social worker; *n* = 2 other)	*Qualitative* Questionnaire; open‐ended questions (changes in perceptions, understanding, empathy since intervention)	Experiences of living with dementia, Alzheimer's disease	*In person* Co‐hosted by Alzheimer's Society; chat led by two moderators	2.5 h book chat Book copies provided 6 weeks ahead of discussion
Prahl et al. (2016)	Sweden	*n* = 1 nursing home	*n* = 12 nursing home staff	*Qualitative* Interviews	To provide an education for nurses to become advisors and to encourage careers in the field of dementia care. Based on cornerstones palliative care (control of symptoms, communication, support next of kin and teamwork) Offers certification for dementia units	*In person* Conducted within a University Lectures, group discussions	Living well with dementia 3 days; staff management 2 days; reflection advice training 2 days and 1 day follow up. Re‐certification every 3 years
Rokstad et al. (2017)	Norway	Community‐based and care homes	*n* = 1795 staff (38% nursing home unit; 20% special care units for people with dementia; 17% sheltered accommodation for people with dementia; 2% day care centres; 20% home‐based nursing care) participated, of which, *n* = 580 completed all assessments. At baseline, 61% auxiliary nurses; 26% registered nurses/social care workers/OT; 10% nurse assistants/housekeeping/dietary personnel/drivers and gardens.	*Quantitative* Longitudinal questionnaire (P‐CAT; psychosocial workplace environmental and job satisfaction) at baseline, 12 months, 24 months and 6 months post‐intervention	Two sets of booklets (*n* = 23): First: general information on learning process, dementia diseases, approaching people with dementia, based on respect for their feelings, personality, behaviour and coping strategies. Second: ethical considerations and how to meet the needs of family carers, promoting meaningful activities, use of validation, reminiscence and music.	*In person* Dementia ABC is coordinated as a national programme by Ageing & Health Written materials, multidisciplinary reflection groups and workshops.	24 months Introduction workshop Baseline – 12 months. Collection of first set of booklets. Self‐run in house discussion groups and a workshop. 12 months—24 months. Collection of second set of booklets. Self‐run in house discussion groups and a workshop. Discussion groups lasted 90 to 120 min and run every second or third week. Two annual workshops
Scerri and Scerri (2019)	Malta	Nursing/residential care homes	*n* = 304 attended the first session; *n* = 218 attended the last session. *n* = 261 staff (*n* = 22 enrolled nurse; *n* = 163 registered staff nurse; *n* = 76 nursing officer/deputy charge nurse) completed pre‐intervention questionnaire *n* = 214 staff (*n* = 24 enrolled nurse; *n* = 132 registered nurse; *n* = 58 nursing officer/deputy charge nurse) completed post‐intervention questionnaire	*Quantitative* Pre‐post‐test questionnaire (ADKS; DAS; CODE; training feedback)	Topics covered: introduction to dementia care and management; access to dementia services; activities for older people with dementia; behavioural issues in caring for individuals with dementia in LTC settings; dementia friendly design and assistive technologies; dementia policy and development	*In person* Training centre where most staff worked Delivered by local experts in dementia and age mental health Questions and discussion at the end of each session	14‐h training programme consisted of 7 × 2‐h sessions
Sheaff et al. (2018)	England	*n* = 23 care homes (*n* = 10 control; *n* = 13 DLC)	Staff across intervention and control care homes People with dementia across intervention and control homes *n* = 4 dementia champions	*Mixed method* Realist evaluation. Completion of DLC intervention; PSDA cycles; DAS; ADQ; SNCW; sickness leave rates; DEM‐QoL/QUALID; DCM; WIB; number of residents with EoL discussions and EoL plans; TEP; number of ambulances call outs and hospital admissions. Interviews with dementia champions; learning facilitators' field notes	*Intervention* Dementia learning facilitators identify one dementia champion per care home. Training covered: nature of dementia, principles of communicating with persons with dementia, PCC, care planning and end‐of‐life care, the Mental Capacity Act and its implications to dementia care, dealing with challenging behaviour and creating and managing organisational change – PDSA cycles Ongoing support to help develop dementia champions' leadership skills and confidence; clarify staff roles in the homes; promote best‐practice in PCC; improve care planning; enhance care environment *Control* Treatment as usual	*Blended* Learning facilitators regularly visited each dementia champion and used networking activities (teleconferences; web‐based forum; monthly team awards; newsletters; annual conference)	Mean participation in intervention 12.3 months (11–14) 8 h multi modal training programme
Su et al. (2021)	Taiwan	*n* = 2 home care agencies	Home care workers; *n* = 140; 70 control and 70 intervention	*Quantitative* Questionnaires baseline, following intervention, 12 weeks follow‐up (DKAS; ADQ; SCIDS)	e‐learning 8 modules: psycho‐behavioural symptom management skills; communication skills; cognitive and emotional assessment; common dementia care problems and care skills; needs assessment and health educational skills for families with dementia; overview of therapeutic activities; care skills for various stages of dementia; oral care for older adults with dementia	*Blended* Intervention: mobile e‐learning, mentor led online social support networking and monthly face‐to‐face mentoring support group meetings. Accessible on mobile phones or tablets One home care service supervisor would act as a mentor to lead 8–10 home care workers. Support daily over the phone or through the group chat function of a free social networking platform. New dementia care information and learning videos uploaded to the social networking platform. Control: usual 8 h lectures.	12‐week intervention. Each e‐learning module 15–20 min. New dementia care information and learning videos were uploaded frequently to the social networking platform to keep home care workers up to date with dementia care knowledge 1 h monthly face‐to‐face support group meetings
Sung et al. (2022)	Taiwan	*n* = 1 home care agency	*n* = 124 home care workers completed the study VR group: *n* = 61 Non‐VR group: *n* = 63	*Quantitative* Cluster randomization control; baseline, end of intervention, 1 month follow‐up Questionnaire (DKAS; ADQ; SCIDS; JSE)	Six dementia care e‐book modules, experience of living with dementia (visual spatial deficits and hallucinations) VR based activity. Discussed care problems during face‐to‐face peer support groups.	*Blended* Intervention: dementia care e‐book modules; dementia VR based activity and peer support group meetings. Peer support groups facilitated by senior peers. Control: dementia care e‐book modules; 1‐h monthly staff meeting	3‐month intervention Instructed to read at least e‐book modules per month. Reminders sent on group chat of a free social networking app weekly to motivate participants to read Two 5‐min videos using VR device and participate in one 50 min discussion 1 h monthly face‐to‐face peer support group meetings 1 h monthly regular staff meeting
Surr et al. (2019)	UK	*n* = 3 care home provider organisations	Training leads, facilitators, staff attending training, managers, residents and their relatives across 3 sites. Two sites comprised two care homes; one comprised one care home.	*Mixed method* Embedded collective case study design Interviews and FGD with dementia training leads, staff, facilitators and managers. Observations (using DCM), WIB, audit of training materials and satisfaction cards with residents and relatives.	General dementia education and training Different and bespoke training implemented across 3 sites	*Mixture* One site implemented a blended approach and two an in‐person format Included self‐directed study via workbooks e‐learning, discussions, role play. exercise, videos, monthly meetings/tutorials, handouts, video clips	
Torres‐Castro et al. (2022)	Mexico	*n* = 7 care homes	*n* = 126 staff (*n* = 57 intervention; *n* = 39 control) *n* = 55 residents (and their relatives) (*n* = 32 intervention; *n* = 23 control)	*Mixed method* Questionnaire: baseline, 12 post intervention) and 24 weeks (follow‐up (MBI; ADQ; NPI‐NH; QoL‐AD; SCIDS) *n* = 12 FGD with residents, family members and staff	Understanding dementia diagnosis, types and clinical assessment, introduction to the international Newcastle cognitive‐behavioural model, team working and organisational psychology elements, psychosocial interventions (including doll therapy), dance‐based psychomotor therapy, reminiscence therapy, PCC (cognitive simulation and life story books), anti‐ Psychotic prescription review. Control: Treatment as usual	*In person* In each care home 12 week implementation period	16‐h (2‐day) staff training –flexible schedules in each care home. Fidelity to the protocol was supervised once a week during 12‐week intervention
Zhao, Liu, et al. (2022)	China	*n* = 10 nursing homes (*n* = 5 intervention; *n* = 5 control)	Intervention: *n* = 101 care home staff (*n* = 59 HCA; *n* = 42 health professionals); T1: *n* = 84; T2: *n* = 74 Control: *n* = 116 care home staff (*n* = 80 HCA; *n* = 36 health professionals); T1: *n* = 96; T2: *n* = 87.	*Quantitative* Multi‐site quasi experimental. Pre‐test post‐test. Questionnaires (SCIDS; DKAS; ADQ; P‐CAT; NPI‐NH) at baseline, post‐ intervention and 3‐months follow up.	DECENT programme: Understanding dementia and dementia‐related issues; PCC; Care communication; Understanding challenging behaviours and management skills; Dementia care in daily living activities; ‘Dementia‐friendly environment; Interaction with families; Care staff's self‐care and development.	*In person* Intervention: sessions by trained facilitators; hand‐outs prior to the sessions and homework Multiple pedagogical strategies: lectures, case study, video clips, reflection and discussion Control: hand‐outs for self‐directed learning	Sessions lasted between 60 and 90 min 1 session weekly over 8 weeks
Zhao, Ding, et al. (2022) and Zhao, Liu, et al. (2022)	China	*n* = 1 nursing home	*n* = 12 healthcare professionals participated (*n* = 7 HCA; *n* = 5 nurses) *n* = 9 finished the post‐intervention assessment; *n* = 5 interviewed (*n* = 3 HCA; *n* = 2 nurses)	*Mixed method* One‐group pre‐post‐test Questionnaire (SCIDS; DKAS; ADQ; P‐CAT; NPI‐NH; feedback on training) Interviews	Same as Zhao, Liu, et al. (2022)	Same as Zhao, Liu, et al. (2022)	Same as Zhao, Liu, et al. (2022)
Zhao et al. (2023)	China	*n* = 5 nursing homes	*n* = 14 nursing home staff (*n* = 5 HCA; *n* = 3 nurses; *n* = 4 managers; *n* = 2 social workers)	*Qualitative* Semi‐structured interviews within 1 week following training completion	Same as Zhao, Liu, et al. (2022)	Same as Zhao, Liu, et al. (2022)	Same as Zhao, Liu, et al. (2022)

Abbreviations: ADKS, Alzheimer's Disease Knowledge Scale; ADL, Activities of Daily Living; ADQ, Approaches to Dementia Questionnaire; CAN, Certified Nursing Assistant; CARES, Connect, Assess, Respond, Evaluate and Share; CCQ, Creative Climate Questionnaire; CHATS, 13‐item forms of a scenario‐based Changing Talk Scale; CODE, Confidence in Dementia Scale; DAD, Disability Assessment for Dementia Scale; DAS, Dementia Attitude Scale; DCM, Dementia Care Mapping; DCW, Direct Care Worker; DECENT, A Culturally Specific Dementia Competence Education for Nursing Home Taskforce; DEMQoL, Dementia Quality of Life Measure; DKAS, Dementia Knowledge Assessment Scale; DLC, Dementia Learning Community; EoL, End of Life; FGD, Focus Group Discussion; HCA, Health Care Assistant; JSE, Jefferson Scale of Empathy; LHW, Lay Health Workers; LPN, Licensed Practical Nurse; LTC, Long‐Term Care; MBI, Maslach Burnout Inventory; NDCWs, Non‐direct Care Workers; NHNAJSQ, Nursing Home Nurse Aide Job Satisfaction Questionnaire; NPI‐NH, Neuropsychiatric Inventory‐Nursing Home Version; OT, Occupational Therapist; P‐CAT, Person‐centred Care Assessment Tool; PCC, Person Centred Care; PDSA, Plan, Do, Study, Act; QoL, Quality of Life; QoL‐AD, Quality of Life‐Alzheimer's Disease Scale; QUALID, Quality of Life in Late Stage Dementia; SCIDS, Sense of Competence in Dementia; SDCS, Strain in dementia scale; SNCW, Satisfaction with Nursing Care and Work Assessment Scale; TEP, Treatment Escalation Plan; VR, Virtual Reality; WIB, Well and Ill‐Being; WRSI, Work‐related Strain Inventory.

^a^ Relates to AWTD intervention only.

^b^ Relates to the intervention for residential care facility staff only.

### 3.2 Intervention Contents

The content of most interventions included input from experts in dementia care, gerontologists, academics, long‐term care consultants, third sector organisations/charities, experts in dementia education, relevant stakeholders within the Ministry of Health and/or from social care staff (incl. Scerri and Scerri 2019; Cooper et al. 2024; Irvine et al. 2012; Inker et al. 2021; Hobday, Savik, Smith, and Gaugler 2010; Hobday, Savik, and Gaugler 2010). People with dementia and their family carers were involved in co‐designing one intervention (Cooper et al. 2024; Kelleher et al. 2022, 2024). One paper reported on a publicly available App developed by Alzheimer's Research UK (Aicken et al. 2021), which was guided by people living with different forms of dementia.

Many interventions comprised an overview of causes of dementia, symptoms and possible needs experienced by people with dementia (Table 2). This included content to understand disease risks, dementia diagnosis, types and/or clinical assessments (incl. Torres‐Castro et al. 2022; Hobday, Savik, Smith, and Gaugler 2010; Hobday, Savik, and Gaugler 2010; Karungi et al. 2022), dispel beliefs and misconceptions (Karungi et al. 2022; Figueiredo et al. 2013), neurological changes associated with dementia (Ehlman et al. 2018), challenging behaviour (Zhao, Ding, et al. 2022; Zhao, Liu, et al. 2022; Scerri and Scerri 2019) and possible unmet social and physical needs (Ehlman et al. 2018). Four interventions sought to stimulate the experience of living with or caring for someone with dementia (Aicken et al. 2021; Inker et al. 2021; Larocque et al. 2014; Sung et al. 2022).

Many interventions included content relating to approaches to caring for and supporting people with dementia and their families (Table 2). These included the tenets and delivery of person‐centred care (Fallahpour et al. 2020; Zhao et al. 2023), communication strategies (Karungi et al. 2022; Hobday, Savik, Smith, and Gaugler 2010; Hobday, Savik, and Gaugler 2010; Inker et al. 2021; Su et al. 2021; Irvine et al. 2012; Rokstad et al. 2017), problem‐solving skills (Figueiredo et al. 2013), managing symptoms and challenging behaviour (incl. Beer et al. 2011; Scerri and Scerri 2019; Rokstad et al. 2017); fostering effective caring relationships (Beer et al. 2011; Chan et al. 2020), delivering meaningful activities (Cooper et al. 2024; Rokstad et al. 2017), specific therapies (Rokstad et al. 2017; Su et al. 2021; Torres‐Castro et al. 2022), assistive technology (Scerri and Scerri 2019) and dementia‐friendly environments (Figueiredo et al. 2013; Scerri and Scerri 2019). One intervention included content to increase community awareness by organising support groups, public campaigns and memory cafes (Karungi et al. 2022).

While some interventions included content on different stages or types of dementia and end‐of‐life care (Sheaff et al. 2018; Su et al. 2021; Prahl et al. 2016; Torres‐Castro et al. 2022), only one intervention focused on late‐stage dementia (Hobday, Savik, and Gaugler 2010) and one incorporated visual hallucinations experienced by people with Lewy body dementia (Sung et al. 2022).

Eleven papers reported on interventions that included content promoting staff wellbeing, peer support and/or self‐care strategies (Cooper et al. 2024; Figueiredo et al. 2013; Inker et al. 2021; Irvine et al. 2012; Karungi et al. 2022; Kelleher et al. 2022, 2024; Rokstad et al. 2017; Zhao, Ding, et al. 2022; Zhao, Liu, et al. 2022; Zhao et al. 2023). Some interventions encompassed topics relating to organisational issues, including staff management, developing leadership skills, teamworking and/or organisational psychology (Sheaff et al. 2018; Prahl et al. 2016; Torres‐Castro et al. 2022). Furthermore, dementia policy and development (Scerri and Scerri 2019), legal aspects relating to dementia care (Chan et al. 2020), ethical considerations to dementia care (Rokstad et al. 2017) and the implications of the Mental Capacity Act^1^ on dementia care (Sheaff et al. 2018) were included in some interventions.

### 3.3 Intervention Delivery Methods

Thirteen papers reported on training delivered in‐person, nine remotely and four used a blended approach. One paper reported on three training sessions: one that employed a blended approach and two delivered in‐person (Surr et al. 2019).

Training ranged from web‐based courses and in‐person discussions (Fallahpour et al. 2020), handouts, group discussions and homework exercises (Figueiredo et al. 2013), to mentorship, lectures and e‐learning (Su et al. 2021), and a smartphone app with virtual reality (Aicken et al. 2021). Some methods of delivery included basic virtual reality, using research members' smartphones placed inside a cardboard headset to view up to three daily scenarios of living with dementia—in a shop, street setting or making refreshments for a visitor (Aicken et al. 2021). Thus, whilst the training was delivered one‐on‐one at the care facility, there was no human interaction as such to deliver the training, which instead was delivered via an app/virtual reality technology on a smartphone. Other innovative methods to raise awareness of dementia included a 2.5 h book chat at a care home in Canada, inviting care home staff to read and discuss the book *Still Alice* (Larocque et al. 2014). Convening as a group and using a book about the topic enabled staff to share their own care experiences about the topic and thus not only learn about dementia, but also connect and share their own experiences within the work setting. In contrast, more information‐based educational programmes such as the Dementia ABC model, also delivered in person, utilized three angles of intervention delivery, including booklets (read in the participants' own time), discussion groups at the care sites, and two annual workshops (Rokstad et al. 2017). Similar information‐based interventions included varying numbers of lessons, ranging from 3 to 52, either delivered in person in the care settings or online on the computer, tablet, or smartphone (Hobday, Savik, Smith, and Gaugler 2010; Hobday, Savik, and Gaugler 2010; Beer et al. 2011; Dobbs et al. 2018; Scerri and Scerri 2019; Inker et al. 2021).

### 3.4 Care Settings and Workforce Groups

Evidence mostly comprised interventions conducted with staff working in one care setting, either care homes (including nursing, residential, long‐term facilities and skilled health facilities) (*n* = 17) or in the community (*n* = 7). The latter was comprised mostly (*n* = 6) of interventions conducted with staff providing home care (Table 2). Most papers reported on interventions (*n* = 14) conducted in care homes that were addressed to mixed cadres of staff, including registered nurses, nurses with managerial responsibilities, health care assistants, allied health professionals, social workers, care home managers/directors, housekeeping staff and/or administrative staff (incl. Beer et al. 2011; Ehlman et al. 2018; Figueiredo et al. 2013). Community‐based interventions were mostly addressed to healthcare/nursing aides (incl. Cooper et al. 2024; Fallahpour et al. 2020; Kelleher et al. 2022), with one addressed to lay health workers (Karungi et al. 2022). Three papers reported findings of interventions conducted with staff working in care homes and community‐based setting(s), including assisted living/sheltered accommodation, day care centres, home‐based nursing care (Rokstad et al. 2017) and community centres (Hobday, Savik, Smith, and Gaugler 2010; Hobday, Savik, and Gaugler 2010; Rokstad et al. 2017; Chan et al. 2020).

### 3.5 Outcomes on Staff Including Knowledge, Competencies and Attitudes

Most papers reported improved knowledge among staff (Table 3) following interventions delivered online, in‐person and by blended approaches. Changes in knowledge were mostly measured by validated tools and/or interviews or focus group discussions (Table 2). In some papers, staff completed ‘knowledge tests’ (Ehlman et al. 2018; Hobday, Savik, Smith, and Gaugler 2010; Hobday, Savik, and Gaugler 2010), video stimulated tests (Irvine et al. 2012) and wrote reflective essays (Chan et al. 2020). Outcomes included greater understanding of the disease, how people and their families may experience dementia, different approaches to care, problem‐solving skills, the importance of delivering person‐centred care and/or how to support/guide staff to provide care. Prahl et al. (2016) found that the intervention facilitated staff to acknowledge their tacit knowledge. Some interventions served as a reminder/confirmation of existing knowledge (Inker et al. 2021; Prahl et al. 2016) and/or provided limited new learning for experienced staff (Surr et al. 2019; Inker et al. 2021). Knowledge gains were present at 12 weeks (Su et al. 2021) and 4 weeks (Sung et al. 2022) following interventions; however, they were not sustained at 3 months follow‐up (Zhao, Liu, et al. 2022).

**TABLE 3:** Grouped key outcomes employed by papers.

References	Key grouped outcomes					
	Knowledge/understanding	Confidence/self‐efficacy/sense of competence	Attitude towards dementia/empathy	Job strain/satisfaction	Changes to practice	Impact on people with dementia and/or family carers
Online						
Aicken et al. (2021)^a^	✔		✔		~	
Cooper et al. (2024)		✘		✘		~
Dobbs et al. (2018)	✔	✔				
Hobday, Savik, Smith, and Gaugler (2010) and Hobday, Savik, and Gaugler (2010)	✔	✔			✔	
Hobday, Savik, and Gaugler (2010)	✔	✔				
Inker et al. (2021)	~		~	~	~	~
Irvine et al. (2012)	✔	~	✔			
Kelleher et al. (2022)	✔	✔	✔		✔	
Kelleher et al. (2024)	✔	✔			✔	✔
In‐person						
Beer et al. (2011)^b^	✔				✔	
Chan et al. (2020)	✔	✔	✔	✔	✔	✔
Fallahpour et al. (2020)				~		
Figueiredo et al. (2013)	✔		✔		✔	✘
Karungi et al. (2022)	✔		✔		✔	~
Larocque et al. (2014)	✔	✔	✔		✔	
Prahl et al. (2016)	✔				✔	
Rokstad et al. (2017)				~	~	
Scerri and Scerri (2019)	✔	✔	✔			
Torres‐Castro et al. (2022)	✔	✘	~	~	✔	~
Zhao, Ding, et al. (2022) and Zhao, Liu, et al. (2022)	✔	✔	✘		✔	✔
Zhao, Liu, et al. (2022)	~	✔	✘		~	✘
Zhao et al. (2023)	✔	✔	✔		✔	
Blended						
Ehlman et al. (2018)	✔		~			
Sheaff et al. (2018)	✔	✔	✘	~	~	~
Su et al. (2021)	✔	~	✔			
Sung et al. (2022)	~	~	~			
Mixture						
Surr et al. (2019)	~	✔	✔		~	~

*Note:* Knowledge/understanding: of dementia; possible needs experienced by people with dementia; how to care for people with dementia; how to train/coach other social care professionals on how to care for people with dementia. Changes to practice: self‐reported; observed; measured changes. Excludes intended changes to practice. Impact on people with dementia and/or family carers: perceived by staff; observed by staff/research teams; reported by people with dementia/family carers. ✔, positive change; ~, mixture of positive and no change; ✘, no change.

^a^ Relates to AWTD intervention only.

^b^ Relates to the intervention for residential care facility staff only.

Many papers report improved staff confidence, competence and/or self‐efficacy in providing care following interventions delivered online, in‐person, or by blended approaches (Table 3). Outcomes included increased confidence while interacting with people with dementia and/or family (Hobday, Savik, Smith, and Gaugler 2010; Hobday, Savik, and Gaugler 2010; Chan et al. 2020; Zhao et al. 2023; Surr et al. 2019), in their skillset (Dobbs et al. 2018; Hobday, Savik, and Gaugler 2010), advocating for change (Kelleher et al. 2022, 2024), and an intervention fostered a greater sense of self‐worth (Figueiredo et al. 2013). Compared to different types of staff, non‐licensed staff (Irvine et al. 2012) and clerical staff (Chan et al. 2020) demonstrated the greatest improvement in self‐efficacy or sense of competence in the dementia care scale, respectively. Findings relating to sustained improvements in staff confidence, competence, and/or self‐efficacy following interventions seemed favourable (Zhao, Liu, et al. 2022; Su et al. 2021; Sung et al. 2022). Sung et al. (2022) reported on a statistically significant increase at 4 weeks follow‐up, which was not observed immediately post‐intervention, suggesting that building a sense of competence may take time. However, not all papers reported improvements (Cooper et al. 2024; Torres‐Castro et al. 2022).

Some evidence suggested improvements in staff attitude and/or empathy (Table 3). Changes in attitudes were mostly quantitatively measured by the approaches to dementia questionnaire (ADQ) and Dementia Attitude Scale (Table 2), and one paper measured empathy with the Jefferson Scale of Empathy (Sung et al. 2022). Improved attitude and empathy were reported (Inker et al. 2021; Ehlman et al. 2018; Irvine et al. 2012; Chan et al. 2020; Scerri and Scerri 2019), with some evidence suggesting these were sustained (Sung et al. 2022; Su et al. 2021). However, not all papers observed improvements (Sheaff et al. 2018; Zhao, Ding, et al. 2022; Zhao, Liu, et al. 2022). Torres‐Castro et al. (2022) found that staff receiving the intervention had similar total scores on the ADQ to the control group; however, they held a statistically significant higher score on the person‐centred sub‐scale, which was sustained 12 weeks post intervention. Qualitatively, staff reported improved attitudes (Larocque et al. 2014; Karungi et al. 2022; Surr et al. 2019), increased reflection on how people with dementia may experience receiving care (Aicken et al. 2021; Kelleher et al. 2022; Zhao et al. 2023; Surr et al. 2019), and a greater appreciation of people with dementia's abilities (Chan et al. 2020; Figueiredo et al. 2013).

Some evidence suggested that interventions, delivered online, in person, or by blended approaches, helped to reduce job strain and/or improve job satisfaction (Table 3). Cooper et al. (2024) observed similar work‐related strain inventory levels across control and intervention groups, and Sheaff et al. (2018) report a statistically significant improvement in the total score of the Satisfaction with Nursing Care and Work Scale within the intervention group. Across types of staff, professional staff, followed by care assistants, observed the greatest improvements in satisfaction (Chan et al. 2020). Three interventions led to statistically significant improvements in some sub‐scales of validated tools, including balancing competing needs (Strain in Dementia Care Scale) (Fallahpour et al. 2020) and personal fulfilment (Maslach Burnout Inventory) (Torres‐Castro et al. 2022). Rokstad et al. (2017) observed increased satisfaction in workload, personal and professional development, demand balanced with qualifications and support, and supervision (psychosocial workplace environment and job satisfaction questionnaire). One intervention led to fewer staff sick days but had no influence on the turnover rate (Sheaff et al. 2018).

Staff who had not been involved in dementia care or who had never received dementia training generally observed greater improvements across outcomes (Chan et al. 2020); however, staff who had completed previous training on dementia continued to improve their knowledge, confidence and attitudes towards dementia (Scerri and Scerri 2019).

### 3.6 Changes to Practice

While not all staff or managers reported changes (Aicken et al. 2021; Inker et al. 2021), 18 papers report on perceived and/or observed changes to practice, which were largely explored and reported in interventions delivered online or in person (Table 3). Outcomes included applying learned approaches, increased communication with families (Zhao et al. 2023), increased teamworking (Prahl et al. 2016; Fallahpour et al. 2020), and/or completing health facility referrals (Karungi et al. 2022). Some interventions led to the perceived increased delivery of person‐centred care (Beer et al. 2011; Chan et al. 2020; Surr et al. 2019; Rokstad et al. 2017), including the delivery of person‐centred activities (Chan et al. 2020) and communication approaches (Surr et al. 2019). However, observation data suggest that person‐centred approaches to communication were not adopted by all staff (Surr et al. 2019). Three papers report on an increase in total scores of the person‐centred care assessment tool (P‐CAT) (Rokstad et al. 2017; Zhao, Ding, et al. 2022; Zhao, Liu, et al. 2022); however, only Rokstad et al. (2017) found evidence of sustained improvements at some study timepoints. The organisational support P‐CAT sub‐scale decreased (Rokstad et al. 2017) or was unchanged (Zhao, Ding, et al. 2022; Zhao, Liu, et al. 2022). Moreover, Sheaff et al. (2018) found no changes in the frequency of end‐of‐life care discussions or care plans.

### 3.7 Impact on People With Dementia and/or Families

Some staff perceived and/or their managers observed that changes to practice positively impacted people with dementia and their families (Surr et al. 2019; Inker et al. 2021; Kelleher et al. 2024; Karungi et al. 2022). Some people with dementia and/or family members reported having enjoyed participating in the activities provided by the intervention (Torres‐Castro et al. 2022) and were satisfied with the care received (Surr et al. 2019). The impact on people with dementia's quality of life was mixed. Questionnaires completed by people with dementia and/or by proxies (staff and/or family carers) suggested limited (Cooper et al. 2024; Torres‐Castro et al. 2022; Sheaff et al. 2018) or some improvement (Cooper et al. 2024) in quality of life. Sheaff et al. (2018) observed improved averages in well and ill‐being scores of people with dementia in half of the care homes within the intervention group, with the other half exhibiting similar or worse levels than the control group. Observational data suggested that people with dementia experienced moderately good levels of well‐being, though this varied across units and people (Surr et al. 2019). Some interventions resulted in increased levels of engagement/interaction among people with dementia living in care homes, though these were not statistically significant (Figueiredo et al. 2013) and/or varied across units (Surr et al. 2019). Regarding the perceived disturbance of behavioural and psychological symptoms, decreasing trends were identified (Cooper et al. 2024; Zhao, Ding, et al. 2022; Zhao, Liu, et al. 2022), and Torres‐Castro et al. (2022) observed a statistically significant improvement at certain study time points. Two papers reported a reduced prescription of drugs (Torres‐Castro et al. 2022; Surr et al. 2019). Sheaff et al. (2018) found that the intervention led to no changes in the number of ambulances called out or emergency hospital admissions.

### 3.8 Quality Ratings

The completed CASP and MMAT checklists (Appendix S1) highlight the following methodological weaknesses for each study design. Few quantitative randomised controlled or non‐randomised trials reported on the adherence to the intervention or whether the intervention was administered as intended. The rationale for employing mixed‐method study designs was often missing, and most qualitative papers did not discuss the relationship between the participants and researcher.

## 4 Discussion

Dementia‐specific training can effectively educate social care staff working in different care settings about core aspects of the condition, improve care delivery and positively affect people living with dementia and their carers and staff. This systematic review comprises literature from 13 countries (primarily from higher‐income settings), evidencing a large variety of dementia‐specific training for social care staff with mostly positive changes and outcomes on staff and, in some cases, service users.

Focusing solely on the basics of dementia in the training provided, thereby excluding various studies focusing specifically on the topics of behavioural management of symptoms or person‐centred communication (e.g., Bielderman et al. 2021; O'Rourke et al. 2022), the included studies showcase a spread of content on disease risks, dementia diagnosis, types and/or clinical assessments, neurological changes associated with dementia, challenging behaviour and possible unmet social and physical needs (Ehlman et al. 2018; Torres‐Castro et al. 2022; Zhao, Ding, et al. 2022; Zhao, Liu, et al. 2022). However, it appears that little attention has been paid to the more advanced stages of dementia, which are of particular importance in care delivery, especially in residential care facilities. It is possible that training on advanced dementia instead is included in generic palliative care training and thus end of life, as for example reported in a narrative review of educational interventions to improve palliative care for people with dementia (Raymond et al. 2013). Considering the different stages of dementia and greater care needs as the condition progresses, it is crucial to include learning about advanced dementia, including losing mental capacity, general reductions in cognition and speech, behavioural changes and how to best provide care. Furthermore, there appears to be little focus on educating staff about the rarer subtypes of dementia, such as Lewy Body dementia, Parkinson's Disease dementia, fronto‐temporal dementias and young‐onset dementia, with some evidence on visual hallucinations in Lewy Body dementia only (Sung et al. 2022). In light of varied symptoms and needs for people with different subtypes (Lima da Silva et al. 2021), training for the social care workforce needs to be modified to be more inclusive of key aspects of dementia.

Despite a lack of focus on some important sub‐areas of dementia, the evidenced dementia training overall was found to positively impact staff knowledge and confidence in delivering care. Considering the highly varied methods of delivery, including book readings, online teaching and apps, in‐person single or multiple events, in different care settings with different supportive or unsupportive infrastructures, it is difficult to ascertain whether a specific type of delivery resulted in the strongest impact. In addition, measurements of impacts varied from qualitative to brief questionnaires to observational data, thus creating challenges in comparing intervention impacts quantitatively. Nevertheless, there is clear potential for wider implementation of dementia‐specific training.

However, receiving dementia training when working in social care is not required for care workers to practice in the UK context, for example. Considering the minimum general training requirements for working in the sector (Hallberg et al. 2014), there is little time to deliver good quality and effective topic‐ and population‐specific training. This is amplified by high rates of staff vacancies in the sector (Skills for Care 2025), leading to often time‐pressured home care visits or care delivery within care settings. Thus, there is little time or opportunity to be released from delivering care in order to improve practice and care delivery (Surr et al. 2020), with supportive management in care organisations equally important to facilitate ongoing training (Surr et al. 2020). This lack of requirement for continued training on dementia, or other population groups, in combination with high staff turnover rates, is likely impacting negatively on the knowledge of staff on how to best care for people living with dementia, including considering their individual needs. Whilst there are some positive and effective examples of dementia‐specific training for the social care workforce, to ensure actual knowledge and practice‐based care delivery outcomes in the sector, this training needs to be implemented on a regular basis and provide social care staff with credits, similar to health care professionals in countries such as the UK (King et al. 2021) or Canada (Sockalingam et al. 2022), and other modes of continued professional learning such as in Rwanda (Nyiringango et al. 2024). Moreover, the sector needs to be transformed into a profession with improved pay, support and recognition for staff. Without these changes, training may reach limited numbers of social care staff and will unlikely have lasting impacts on their care delivery and thus, ultimately, the people with dementia being cared for.

Considering the ultimate goal of improving the well‐being of and care for people with dementia, limited evidence has shown how training can directly benefit people with dementia and carers. Findings on impacting people with dementia and carers were mixed (Cooper et al. 2024; Torres‐Castro et al. 2022; Sheaff et al. 2018) with too few studies to draw definitive conclusions. Instead, there is an indication rather than give an indication of the positive trickle‐down effect from improved staff knowledge to care delivery to the service user. It is possible that evidence is limited due to the more time‐intensive nature of data collection when using the impact on service user well‐being as an additional outcome, likely requiring several months since training delivery to genuinely measure impact. However, to ensure that different training interventions become integrated into NICE guidance on dementia (or the equivalent in countries other than the UK), and part of standardised social care training, where it exists, future research and implementations need to report on the impact of training on people with dementia and carers where relevant.

## 5 Limitations

Whilst this systematic review benefits from having searched five evidence bases, with each abstract and full text screened by at least two research team members, there are some limitations to the existing evidence included in this review. Inclusion was restricted to studies published in English and German, with only English studies relevant for the final included number of studies. This may have restricted possible evidence from non‐English speaking countries. All evidence, except for three studies, has emerged from high‐income countries, with a predominance of research from the US and the UK (12 papers in total). Whilst health care systems and the availability of services vary between high‐income countries and within these, they are more closely aligned than to health care infrastructures in lower‐ and middle‐income countries (LMICs). Health care services are often limited, and access is further impeded for most due to financial barriers and lack of health insurance or universal health care (Barasa et al. 2021; Giebel et al. 2022). Given these restrictions to health care settings in LMICs, social care services mostly fail to exist, with care needing to be provided by family members (Breuer et al. 2021). This might explain the lack of evidence that has emerged from LMICs, except for one study each from China, Mexico and Uganda. In light of ‘limited’ to non‐existent social care provision across these countries, training for the social care workforce, as evidenced in high‐income countries such as the UK and the US, might benefit from being adapted to unpaid carers and thus family members in LMICs, who are the care workforce.

## 6 Conclusions

There is growing evidence as to the varied types of dementia training for the adult social care workforce and its positive impacts on staff knowledge. However, the content of existing and evidenced training programmes lacks a focus on rarer subtypes of dementia and advanced stages of dementia, with limited evidence on the impact of training on people with dementia and their carers. Whilst training therefore should be updated to be more inclusive of diverse aspects of dementia, successful training programmes need to be implemented on a larger scale to be accessible and impactful to social care workers, not only in settings that tested these interventions, but to roll out training within and across different countries. This would directly address a substantial limitation of the social care workforce in the UK (Skills for Care 2025) and can aid in developing a social care workforce in LMICs where social care is often limited and underdeveloped.

## Conflicts of Interest

The authors declare no conflicts of interest.

## Supporting information


**Appendix S1:** jan70288‐sup‐0001‐AppendixS1.docx.

## Data Availability

The authors have nothing to report.

## Endnotes

^1^

https://www.legislation.gov.uk/ukpga/2005/9/contents.
