# Supplementary material for: General Dementia Training for the Social Care Workforce: A Systematic Review
Source: J Adv Nurs. 2025 Oct 21;82(6):5790–812. doi: 10.1111/jan.70288 (PMC13176697; doi:10.1111/jan.70288)
Supplement: Supplementary file 1 — Appendix S1: jan70288‐sup‐0001‐AppendixS1.docx. [file JAN-82-5790-s001.docx]

**Completed Mixed Methods Appraisal Tool (Hong et al., 2018) for included papers with quantitative and mixed methods study designs**

| **References** | | **Responses** | | | | | | | **MMAT methodological criteria** |
| --- | --- | --- | --- | --- | --- | --- | --- | --- | --- |
| *Quantitative randomised controlled trials* | | S1 | S2 | 2.1 | 2.2 | 2.3 | 2.4 | 2.5 | **S1** Are there clear research questions?; **S2** Do the collected data allow to address the research questions?; **2.1** Is randomization appropriately performed?; **2.2** Are the groups comparable at baseline?; **2.3** Are there complete outcome data?; **2.4** Are outcome assessors blinded to the intervention provided?; **2.5** Did the participants adhere to the assigned intervention? |
|  | Su et al., 2021 | Y | Y | N | N | Y | N | CT |  |
|  | Sung et al., 2021 | Y | Y | Y | Y | Y | Y | CT |  |
|  | Cooper et al., 2024 | Y | Y | Y | Y | N | Y | Y |  |
|  |  |  |  |  |  |  |  |  |  |
| *Quantitative non-randomised trails* | | S1 | S2 | 3.1 | 3.2 | 3.3 | 3.4 | 3.5 | **S1** Are there clear research questions?; **S2** Do the collected data allow to address the research questions?; **3.1** Are the participants representative of the target population?; **3.2** Are measurements appropriate regarding both the outcome and intervention (or exposure)?; **3.3** Are there complete outcome data?; **3.4** Are the confounders accounted for in the design and analysis?; **3.5** During the study period, is the intervention administered (or exposure occurred) as intended? |
|  | Zhao, Li & Ding, 2022 | Y | Y | Y | Y | Y | N | CT |  |
|  | Scerri and Scerri, 2019 | Y | Y | Y | Y | N | Y | CT |  |
|  | Irvine et al., 2013 | Y | Y | Y | Y | Y | N | CT |  |
|  | Fallahpour et al., 2020 | Y | Y | Y | Y | N | N | CT |  |
|  | Ehlman et al., 2018 | Y | Y | N | Y | Y | N | CT |  |
|  | Rokstad et al., 2016 | Y | Y | Y | Y | N | Y | CT |  |
| *Mixed methods* | | S1 | S2 | 5.1 | 5.2 | 5.3 | 5.4 | 5.5 | **S1** Are there clear research questions?; **S2** Do the collected data allow to address the research questions?; **5.1** Is there an adequate rationale for using a mixed methods design to address the research question?; **5.2** Are the different components of the study effectively integrated to answer the research question?; **5.3** Are the outputs of the integration of qualitative and quantitative components adequately interpreted?; **5.4** Are divergences and inconsistencies between quantitative and qualitative results adequately addressed?; **5.5** Do the different components of the study adhere to the quality criteria of each tradition of the methods involved? |
|  | Beer et al., 2011 | Y | Y | N | Y | Y | Y | N |  |
|  | Figueiredo et al., 2013 | Y | Y | N | N | N | Y | N |  |
|  | Sheaff et al., 2018 | Y | Y | Y | Y | Y | Y | Y |  |
|  | Chan et al., 2020 | Y | Y | Y | Y | Y | Y | Y |  |
|  | Zhao et al., 2022 | Y | Y | N | Y | Y | Y | Y |  |
|  | Dobbs et al., 2018 | Y | Y | N | N | Y | Y | N |  |
|  | Hobday, Savik & Gaugler, 2010 | Y | Y | N | Y | Y | Y | N |  |
|  | Hobday et al., 2010 | Y | Y | N | Y | Y | Y | N |  |
|  | Kelleher et al., 2024 | Y | Y | Y | Y | Y | Y | Y |  |
|  | Torres-Castro et al., 2022 | Y | Y | N | N | N | N | N |  |
|  | Inker et al., 2021 | Y | Y | N | Y | Y | Y | Y |  |
|  | Surr et al., 2019 | Y | Y | Y | Y | Y | Y | Y |  |
|  | Kelleher et al., 2022 | Y\| | Y | N | N | N | Y | Y |  |

**Key**: Y – yes; N- No; CT – Can’t tell

**Completed CASP checklist (Critical Appraisal Skills Programme, 2024) for papers with qualitative study designs**

| **References** | | **Responses** | | | | | | | | | | **CASP methodological criteria** |
| --- | --- | --- | --- | --- | --- | --- | --- | --- | --- | --- | --- | --- |
| *Qualitative* | | 1 | 2 | 3 | 4 | 5 | 6 | 7 | 8 | 9 | 10 | **1** Was there a clear statement of the aims of the research?; **2** Is a qualitative methodology appropriate?; **3** Was the research design appropriate to address the aims of the research?; **4** Was the recruitment strategy appropriate to the aims of the research?; **5** Was the data collected in a way that addressed the research issue?; **6** Has the relationship between researcher and participants been adequately considered?; **7** Have ethical issues been taken into consideration?; **8** Was the data analysis sufficiently rigorous?; **9** Is there a clear statement of findings?; **10** How valuable is the research? |
|  | Zhao et al., 2023 | Y | Y | Y | Y | Y | Y | Y | Y | Y | Y |  |
|  | Aicken et al., 2021 | Y | Y | Y | Y | Y | N | Y | N | Y | Y |  |
|  | Karungi et al., 2022 | Y | Y | Y | Y | Y | N | Y | N | Y | Y |  |
|  | Prahl et al., 2016 | Y | Y | Y | Y | Y | N | Y | Y | Y | Y |  |
|  | Larocque et al., 2014 | Y | Y | N | Y | N | N | CT | N | Y | Y |  |

**Key**: Y – yes; N- No; CT – Can’t tell
